# Characterizing Immunoglobulin Repertoire from Whole Blood by a Personal Genome Sequencer

**DOI:** 10.1371/journal.pone.0075294

**Published:** 2013-09-13

**Authors:** Fan Gao, Edwin Lin, Yaping Feng, William J. Mack, Yufeng Shen, Kai Wang

**Affiliations:** 1 Zilkha Neurogenetic Institute, University of Southern California, Los Angeles, California, United States of America; 2 Department of Systems Biology, Columbia University, New York, New York, United States of America; 3 Department of Biomedical Informatics, Columbia University, New York, New York, United States of America; 4 JP Sulzberger Columbia Genome Center, Columbia University, New York, New York, United States of America; 5 Department of Psychiatry, University of Southern California, Los Angeles, California, United States of America; 6 Current address: Waksman Genomics Core Facility, Rutgers University, Piscataway, New Jersey, United States of America; University of California, Irvine, United States of America

## Abstract

In human immune system, V(D)J recombination produces an enormously large repertoire of immunoglobulins (Ig) so that they can tackle different antigens from bacteria, viruses and tumor cells. Several studies have demonstrated the utility of next-generation sequencers such as Roche 454 and Illumina Genome Analyzer to characterize the repertoire of immunoglobulins. However, these techniques typically require separation of B cell population from whole blood and require a few weeks for running the sequencers, so it may not be practical to implement them in clinical settings. Recently, the Ion Torrent personal genome sequencer has emerged as a tabletop personal genome sequencer that can be operated in a time-efficient and cost-effective manner. In this study, we explored the technical feasibility to use multiplex PCR for amplifying V(D)J recombination for IgH, directly from whole blood, then sequence the amplicons by the Ion Torrent sequencer. The whole process including data generation and analysis can be completed in one day. We tested the method in a pilot study on patients with benign, atypical and malignant meningiomas. Despite the noisy data, we were able to compare the samples by their usage frequencies of the V segment, as well as their somatic hypermutation rates. In summary, our study suggested that it is technically feasible to perform clinical monitoring of V(D)J recombination within a day by personal genome sequencers.

## Introduction

Over 25 years ago, Susumu Tonegawa won the Nobel Prize in Physiology & Medicine for discovering the genetics behind V(D)J recombination, which refers to the genomic rearrangement of variable (V), diversity (D), and joining (J) gene segments separated by highly variable junction regions [1], [2]. In human genome, the immunoglobulin (Ig) loci contain many different V, D and J segments, which are subject to rearrangement process during early lymphoid differentiation. As a result of random V(D)J recombination, our body can produce enormous amounts of immune cells each with a different Ig gene (mainly B cells). Together with somatic hypermutation, the immune system can adapt to foreign elements and produce antibody molecules to target/neutralize antigens from bacteria, viruses, parasites and dysfunctional cells such as tumor cells. Of note, several lymphoid malignancies in humans are the direct results of monoclonal expansion of a specific B-cell clone, so that the vast majority of B cells have identical V(D)J recombination in patients with lymphoid malignancies [3].

Despite its importance in human disease and health, conventional methods to measure V(D)J recombination have several limitations to prevent detailed characterization of the immune repertoire. Many earlier methods, such as restriction enzyme digestion followed by Southern blotting or sizing of polymerase chain reaction (PCR) products from Ig loci, were developed as simple measures of the clonality of B cells, but they are too coarse to reveal the intra-clonal heterogeneity. Later approaches, such as multi-parameter flow cytometry, spectrotyping, or custom-designed real-time PCR assays, are more quantitative and offer higher resolution, but these methods are labor intensive and are unable to offer sequence-level insights as to the exact V(D)J recombination patterns in patients. Given the enormous amounts of information content embedded within the immune repertoire, sequence-level examination is expected to offer the most detailed characterization of V(D)J recombination in human subjects.

With the development of massively parallel sequencing technologies, it is now feasible to assay V(D)J recombination by next-generation sequencing, as a means to exhaustively profile the immune repertoire in human subjects. One of the first such studies, published in late 2009, measured and clinically monitored human lymphocyte clonality by massively parallel pyrosequencing using the Roche 454 sequencers [4]. In their study, DNA was isolated from blood, and a series of redundant primers was used to amplify IgH locus, and the resulting mixtures of amplicons were sequenced by 454 sequencer. The advantage of using the 454 sequencer was its ability to generate longer sequencing reads that potentially covers V(D)J recombination junction points. This proof-of-concept study demonstrated the technical feasibility to monitor malignancy by sequencing peripheral blood. Another study also used similar techniques to reveal a complex pattern of dynamic relationships among human T cell subsets [5]. These studies relied on the 454 sequencer, due to its capability to generate longer sequencing reads, which are more likely to cover the V(D)J recombination junction points. However, other investigators have focused on Illumina Genome Analyzer that generates only ∼50 bp reads. For example, a group has developed a short-read assembly strategy to first assemble 50 bp sequences and then sample the CDR3 diversity in human T lymphocytes from peripheral blood [6], [7]. The data analysis involved in such strategy is much less straightforward and may not be as deterministic as 454 sequencers, but it has the advantage of much higher throughput and perhaps more readily accessible.

Given the limitations of previous studies, our goal is to evaluate whether a new generation of personal genome sequencers can be used to interrogate V(D)J recombination within a relatively short period of time (within one day). Our study differs in several major aspects: First, instead of relying on flow cytometry or magnetic beads to isolate B cell populations from peripheral blood, we attempted to assay DNA extracted directly from whole blood. Second, we used genomic DNA rather than mRNA, as the extraction of genomic DNA from blood is technically more straightforward and genomic DNA is far less likely to be degraded under clinical storage conditions. Third, we evaluated the usage of Ion Torrent sequencer, an integrated semiconductor device that performs non-optical DNA sequencing of genomes with a turnaround time within hours [8]. It has slightly longer average length of sequencing reads (typically ∼200 bp) than the Illumina sequencers used in previous studies, yet with much lower throughput (typically 1 million reads per run). We also note that the new Illumina MiSeq platform has similar characteristics as Ion Torrent. However, the turnaround time of personal genome sequencers has been significantly shortened compared to Roche 454 or previous generation of Illumina sequencers. Despite slightly shorter reads than Roche 454, these reads may be long enough to interrogate V(D)J recombination directly without *de novo* assembly.

To evaluate our approach, we tested it on blood samples collected from patients with meningiomas − the most common brain tumors in the United States, and compared the sequence data between patients with benign (grade I), atypical (grade II) and malignant (grade III) tumors. Meningioma arises from the membranous layers surrounding the central nervous system, thus it is not subject to blood-brain barrier. Many previous studies have already reported the presence of both humoral [9], [10] and cellular [11], [12] immune responses in patients with meningiomas. Indeed, one previous study has proposed that frequent antibody response against specific antigens in benign meningiomas can serve as diagnostic targets [9]. Therefore, meningiomas are well suited as initial targets to test the technical feasibility of the technology, with the added value of investigating immune differences between tumor subtypes.

## Results

### Multiplex PCR Reactions to Explore V(D)J Recombination on Igh Locus

**Figure 1:**
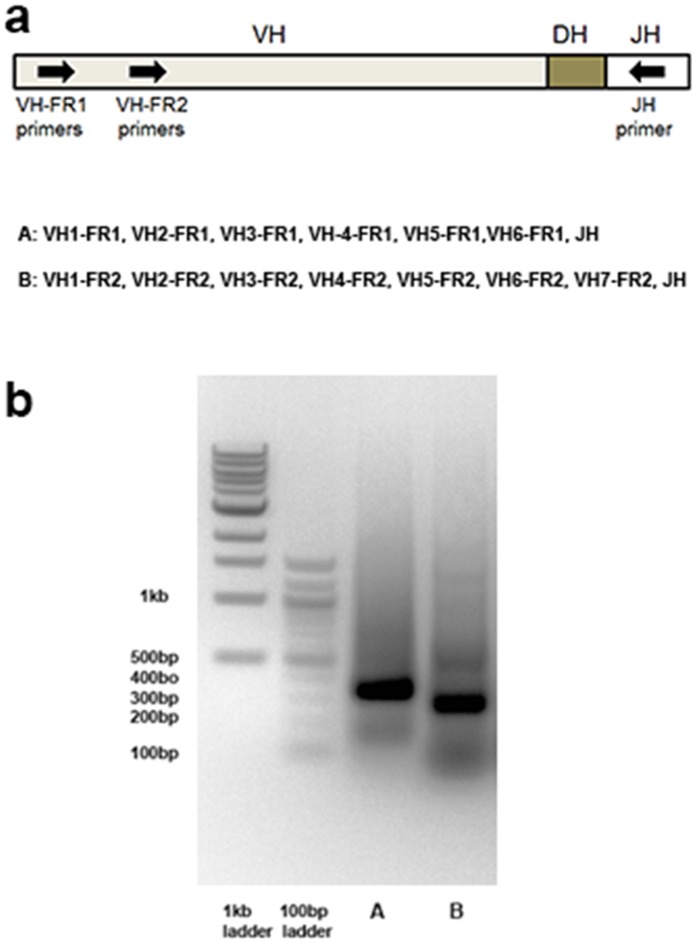
Multiplex PCR reactions to amplify IgH V(D)J recombination on genomic DNA extracted from an EBV-transformed lymphoblastoid cell line (monoclonal positive control). The combinations of two different PCR primer sets (A, B) are also shown in Figure 1a. Predominant bands are observed for pools A & B with sizes around 330 bp and 280 bp (Figure 1b).

Previous BIOMED2 study [13] has evaluated PCR primers for detection of clonally rearranged immunoglobin genes. We followed the protocol and used the same sets of PCR primers (**Table 1**) to perform multiplex PCR reactions to capture rearrangement of immune repertoire in the IgH locus. In particular, we used two pools of PCR primer sets to amplify the DNA fragments between different framework (FR) regions and J_H_ region (**Figure 1a**). To evaluate the effectiveness of the employed PCR protocol, we first tested multiplex PCR amplification on an EBV-transformed lymphoblastoid cell line, which served as a monoclonal positive control. Interestingly, DNA electrophoresis revealed single predominant bands for both V_H__FR1– J_H_ and V_H__FR2– J_H_ pools with predominant fragment sizes around 330 bp and 280 bp, respectively (**Figure 1b**). For a fully rearranged IgH locus, the genomic spans from V_H_ FR1 to J_H_ are between 309 bp and 341 bp, depending on the exact V_H_ subtypes, whereas fragments between V_H_ FR2 and J_H_ are ∼ 50 bp shorter (**Table 1**). Thus the sizes of the amplified DNA fragments from the two primer pools indicate that they are indeed products from V(D)J recombination, most likely reflecting monoclonal expansion for this lymphoblastoid cell line.

**Table 1:** PCR primers for the IgH locus based on the BIOMED2 primer sets.

Primer ID	Primer Sequence	Relative position to RSS*
JH	CTTACCTGAGGAGACGGTGACC	+57
VH1-FR1	GGCCTCAGTGAAGGTCTCCTGCAAG	−252
VH2-FR1	GTCTGGTCCTACGCTGGTGAAACCC	−284
VH3-FR1	CTGGGGGGTCCCTGAGACTCTCCTG	−256
VH4-FR1	CTTCGGAGACCCTGTCCCTCACCTG	−256
VH5-FR1	CGGGGAGTCTCTGAAGATCTCCTGT	−255
VH6-FR1	TCGCAGACCCTCTCACTCACCTGTG	−263
VH1-FR2	CTGGGTGCGACAGGCCCCTGGACAA	−192
VH2-FR2	TGGATCCGTCAGCCCCCAGGAAAGG	−190
VH3-FR2	GGTCCGCCAGGCTCCAGGGAA	−189
VH4-FR2	TGGATCCGGCAGCCCGCCGGGAAGG	−188
VH5-FR2	GGGTGCGCCAGATGCCCGGGAAAGG	−190
VH6-FR2	TGGATCAGGCAGTCCCCATCGAGAG	−194
VH7-FR2	TTGGGTGCGACAGGCCCCTGGACAA	−192

* Relative position is calculated from 5′end of the primer sequence to the involved RSS (recombination signal sequence).

**Figure 2:**
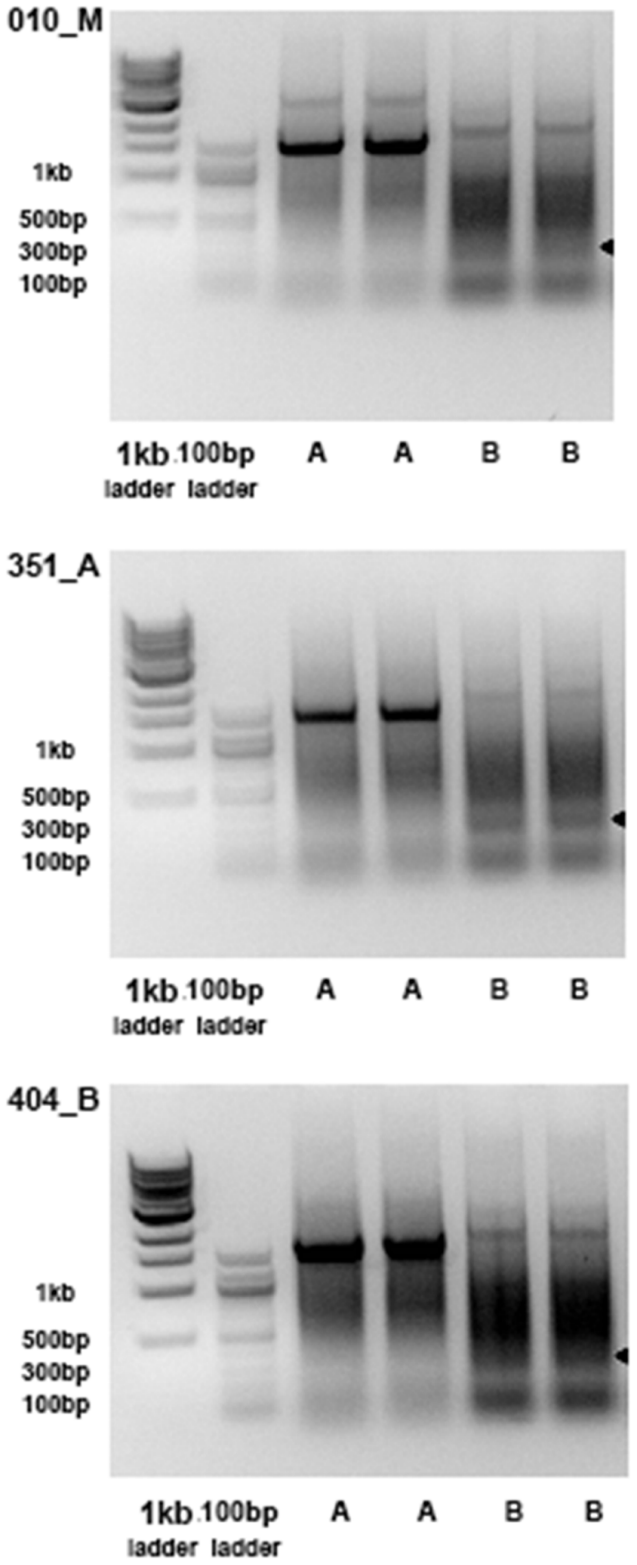
Two pools (A, B) of multiplex PCR reactions to amplify IgH V(D)J recombination on genomic DNA extracted from blood samples of three patients with different grades of meningiomas (malignant –010_M, atypical –351_A, benign –404_B). The bands with size around 280(indicated in arrows) were excised and purified for Ion Torrent sequencing.

Inspired by the initial test run on the lymphoblastoid cells, we applied the multiplex PCR reactions on genomic DNA extracted directly from whole blood samples of three patients affected with benign (grade I), atypical (grade II) or malignant (grade III) meningiomas, respectively. Unlike single predominant bands observed for lymphoblastoid cell line, DNA electrophoresis revealed multiple bands for both primer pools (**Figure 2**). Of note, without isolating mature B cells, the collected genomic DNA may come from B cells with fully rearranged, partially rearranged and intact IgH locus, as well as from other blood cell types. Based on results on positive controls, we hypothesized that the ∼280 bp bands in V_H_-FR2– J pool (**Figure 2**) may have the fully rearranged fragments, thus we excised and purified the bands for next-generation sequencing.

### Clonality of Immune Repertoire Revealed by V Gene Usage

The raw FASTQ files from Ion Torrent sequencer were mapped by BWA-SW [14] against hg19 genome assembly (patched by replacing IgH sequences using the one from IMGT [15]) or aligned against human_gl_V, human_gl_D and human_gl_J libraries from IgBLAST [16]. Our sequencing run pooled additional amplicons (in addition to IgH) from the same sample, so relatively small fraction of the reads can be mapped to the IgH region. The BWA-SW mapping results of the three meningioma samples revealed that between 15.0% and 38.2% of the reads can be mapped to V_H_ regions (**Table 2**). We however noted that only very small fractions of reads (0.4% to 0.5%) can be mapped to V-D or V-D-J junction points. In a parallel analysis using the newly available IgBLAST tool, we also observed that the malignant sample had a higher percentage of V_H_ containing reads (11.4%) compared to atypical or benign ones (4.9% and 5.6%, respectively, **Table 3**). We also noted that 3% to 7% of the reads contained V-D, V-J or V-D-J junctions. Thus, based on both bioinformatics pipelines, V-D-J junction reads were not the predominant form of reads, possibly due to sample processing procedure to sonicate DNA fragments and the relatively short sequence reads from the Ion Torrent sequencer (see Discussion below).

**Table 2:** Summary of BWA-SW analysis of sequencing data from Ion-Torrent PGM.

Sample ID	% of reads unassigned	% of reads uniquely assigned to V_H_ segment	% of reads uniquely assigned to V_H_-D_H_ segment	% of reads uniquelyassigned to V_H_-D_H_-J_H_segment	Total number of reads from IGH targeted sequencing
404_B(benign)	84.5%	15.0%	0.2%	0.3%	294911
351_A(atypical)	79.1%	20.4%	0.2%	0.3%	71000
010_M(malignant)	61.4%	38.2%	0.2%	0.2%	19987

**Table 3:** Summary of IgBLAST analysis of sequencing data (with focus on the V gene).

Sample ID	% of readsunassigned	% of reads containingV_H_ only	% of reads containingV_H_-D_H_ junction only	% of reads containingV_H_-J_H_ junction only	% of reads containingV_H_-D_H_-J_H_ junction
404_B(benign)	79.5%	5.6%	3.5%	4.0%	7.4%
351_A(atypical)	83.2%	4.9%	3.2%	2.8%	5.9%
010_M(malignant)	75.4%	11.4%	4.6%	5.0%	3.6%

**Figure 3:**
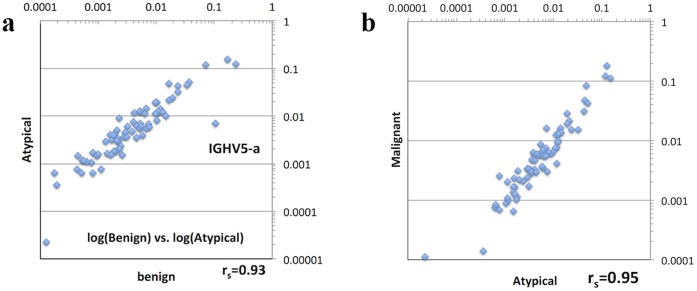
Scatter plots of V segment usage frequencies between benign, atypical and malignant samples based on Ion Torrent sequencing data. The IgHV5-a segment is represented in 10% of the IgH reads in the benign sample, yet <1% in the atypical and malignant samples.

We hypothesized that more severe tumors (grade II and III) may have higher clonality in IgH populations, due to increased humoral immune responses. To test this, we performed correlation analysis to compare the V segment usage frequencies between three tumor subtypes using BWA-SW mapping result (**Figure 3**). However, we did not find apparent difference between the tumor subtypes; that is, V segments that tend to be used more in one tumor type also tend to be used more often in another tumor type. Indeed, the Pearson’s correlation coefficients were greater than 0.9 in each pairwise comparison. Recognizing that we have evaluated merely three samples, our results should be considered as descriptive, and we caution that a definitive conclusion cannot be drawn. However, we also observed a notable exception: the usage of a particular V_H_ gene, IgHV5-a, was sharply dropped in atypical and malignant samples compared to the benign one (**Figure 3**). Interesting to note, although the total fractions of assigned V segment reads from IgBLAST were different from BWA-SW, IgBLAST analysis also showed that IgHV5-a was preferentially used in the benign sample: the fractions of reads containing IgHV5-a in benign, atypical and malignant samples were 0.856%, 0.007% and 0.005%, respectively. Therefore, results from two analytical pipelines are largely concordant, despite different sensitivity to assign reads.

### Analysis of Somatic Hypermutation in Different Meningioma Samples

**Figure 4:**
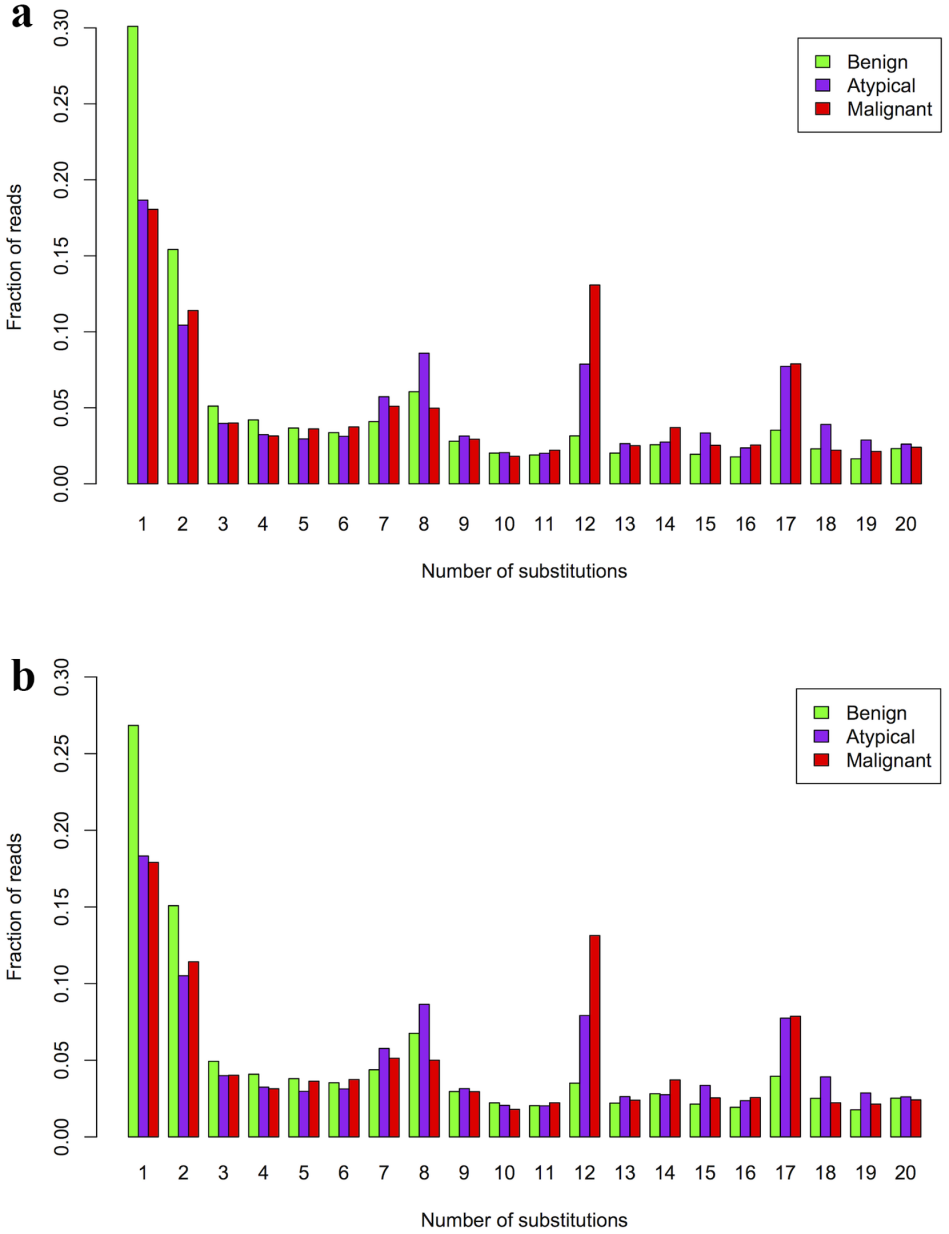
Comparison of somatic hypermutations in reads from IgH regions in three tumor subtypes. The benign tumor tends to have less somatic hypermutations compared to atypical and malignant samples. (a) analysis on all available reads; (b) analysis after excluding IgHV5-a.

Given the availability of individual sequence reads, we next performed comparative analysis on somatic hypermutations between three tumor subtypes. Following activation of naïve B cells, the IgH locus experiences a highly accelerated rate of somatic mutation (increased by a factor of 10^5^ to 10^6^), so that the mutation rate can reach 1 per 1,000 for each V gene base pair per cell division, further diversifying the immune repertoire beyond the genetic makeup of IgH. However, one potential confounding factor is that Ion Torrent sequencer itself may introduce sequencing errors, with observed error rate of 1.5% [17] and 1.7% [18] in different studies. Nevertheless, it is reasonable to assume that the same error rates will apply for all samples that were sequenced at the same time under the same sample preparation procedure. From the set of sequencing data, we inferred the number of somatic hypermutations per sequencing read, and compared the distribution between benign, atypical and malignant samples. Although the overall distribution appeared to be largely similar, we found apparent differences for reads with a small number of mutations. Compared to atypical or malignant meningioma, the benign tumor sample had higher fraction of reads with only one or two somatic hypermutations (**Figure 4a**). We acknowledge that a 1 bp difference could be due to germline variants, but larger number differences in the same reads are more likely to be due to somatic hypermutations. This observation may indicate that more hypermutations tend to be accumulated in more severe cancer subtypes, but our sample size is not enough to make a definitive conclusion. Therefore, we report the results as interesting observations only.

We next attempted to address an alternative possibility that the observed differences in somatic hypermutation rates may be due to difference in V_H_ segment usage, given that IgHV5 is differentially used in the benign tumor in comparison to atypical/malignant samples (**Figure 3**). To test this, we removed the IgHV5 regions from the somatic hypermutation analysis, yet we still obtained similar set of results (**Figure 4b**). Therefore, our analysis demonstrated that there might be differences in rates of somatic hypermutations between different tumor samples. Again we caution that this hypothesis requires much larger sample sizes for further validation.

## Discussion

In this study, we evaluated the technical feasibility of using Ion Torrent personal sequencers to profile the immune repertoire, particularly the IgH locus, from whole blood. Compared to previous studies, our study did not separate B cells, used DNA rather than RNA, applied standard multiplex PCR reactions, and used a sequencer with one-day turnaround time including data analysis. We demonstrated that it is technically feasible to use a personal genome sequencer to interrogate V(D)J recombination. However, the current turnaround time of a personal genome sequencer is still not quick enough for this approach to be considered as a point-of-care test, thus further reduction of turnaround time is necessary. In addition to evaluation of feasibility, we also made preliminary observation that IgH hypermutation rates differ between patients with different severity of the disease.

There are several technical details worth noting. First of all, unlike all previous studies, our goal is not to interrogate patients with lymphoid malignancies who already have clonal expansion of B-cell populations, but to evaluate patients with meningiomas who have humoral immune responses. Therefore, besides the ability to evaluate technology, we also attempted to address an interesting question whether patients with brain tumor differ in immune responses. Due to small sample size, we cannot make a definitive conclusion. Second, to simulate the situation of B-cell clonal expansion, we have assayed a lymphoblast cell line, and confirmed the efficiency of the BIOMED-2 primer sets on samples with monoclonal expansion of B cells. We believe that this step is highly important for our selection of bands to excise in the sequencing experiments. Third, we obtained only ∼150 bp average read length from the Ion Torrent sequencer. Additionally, due to technical limitations, our sample preparation procedure includes a step to sonicate DNA fragments for the Ion Torrent run. Unfortunately, the vast majority of reads do not cover the entire V-D-J junction, complicating our analysis. With the development of sequencers that can generate longer fixed-length reads such as Illumina MiSeq (2×300 bp, 15 Gb throughput) or HiSeq 2500 (2×250 bp, 300 Gb throughput), this problem may be better addressed by different personal genome sequencers in the future. Fourth, the blood samples that we used were previously frozen samples rather than freshly collected blood. Since DNAs are far more stable than RNAs, our results demonstrated that frozen blood samples could yield reliable results, which may not be attainable using RNA-based procedures. Considering that DNAs are much easier to process than RNAs, we expect that DNA-based approaches will be more likely adopted in clinical settings.

One interesting observation from our study is that the benign sample shows different usage statistics of V_H_ segments and has smaller somatic hypermutation rates, in comparison with the atypical and malignant samples. For the former observation, although the sequenced DNA fragments may include both targeted V(D)J recombination events and contaminations (captured V_H_ segments only account for 15% to 38% of total reads), the data collected from meningioma patients with different grade tumors revealed increased portion of V_H_-segment reads in the total reads associated with malignancy of meningiomas. Such an increase may reflect clonal expansion in malignant tumor. For the latter observation, intuitively one would expect to see more frequent somatic hypermutations with increased severity of diseases, as a result of the immune system trying to adapt to tumor antigens. Nevertheless, we still wish to stress that our study is a pilot study and the observations are only restricted to three samples, thus any conclusion requires further validation by studies with larger sample size.

We expect that personal genome sequencers will increasingly find their usage in clinical monitoring of patients and help guide the selection of therapeutic regimens. The capability to generate sequence data within days greatly helps clinical applications. In our study, the use of whole blood rather than isolated B cells, the use of DNA rather than RNA, and the use of Ion Torrent sequencers are the keys to a relatively short turnaround time. Therefore, with further optimization of protocols, this approach may represent a viable option for clinical monitoring of patients with lymphoid malignancies or other types of malignancies. Finally, in the past decades, numerous biobanks with enormous collections of frozen blood have been established and served as resources to study the genetics of human diseases [19]–[22]. Revisiting these resources to decode the embedded immunogenomic information using the latest sequencing platform may provide novel insights into pathogenesis that were not considered in typical genetic studies.

## Materials and Methods

### Sample Collection

We collected peripheral blood from patients with meningiomas from the USC Brain Tumor Bank. All samples have extensive phenotype information, including available consistency data and brain perfusion data (an advanced MRI imaging technique specifically used at USC that shows the blood flow patterns around tumor tissues) to facilitate interpretation of results, especially clonality measures. The study was approved by the Institutional Review Board at the University of Southern California. Informed written consent was obtained for all participants prior to undergoing surgical tumor resection. All patients presenting to Los Angeles County – USC Medical Center or Keck Hospital of USC with benign, atypical or malignant neoplasms of the brain were eligible participants.

### Multiplex PCR Reactions

We directly extracted genomic DNA from frozen whole blood using Qiagen blood DNA extraction kit, without isolating specific cell populations. DNA concentrations were determined by a Nanodrop spectrophotometer. To amplify V(D)J recombination in IgH locus, we followed previous studies and used the BIOMED-II primer sets [13]. We used EMD KOD Hot-Start PCR reaction kit for amplification with 100 ng genomic DNA, 5 µL 10X reaction buffer, 5 µL dNTP mix (2 mM each), 3 µL MgSO4 (25 mM), 2 µL of primer mix (10 µM each), 1 µL of KOD DNA Polymerase in total 50 µL reactions. The PCR reactions started with denaturing at 95°C for 2 minutes, followed by 40 cycles of amplifications with 95°C for 20 seconds, 65°C for 10 seconds, 70°C for 1 minute, and finally 70°C for 2 minutes for product repair. Genomic DNA from EBV-transformed lymphoblastoid cells was used as a positive control for detecting V(D)J recombination. Amplified PCR products were visualized using DNA electrophoresis gel. The bands with expected size were excised and purified using Qiagen MinElute gel Extraction kit for sequencing.

### Generation of Sequencing Data

We used the Ion Torrent 316 chip with the 200 bp kit for generation of sequencing data on the Ion Torrent sequencer. Briefly, we followed the manufacturer’s recommended library construction procedures, sonicated the raw PCR products into ∼250 bp fragments and then added adaptors for next-generation sequencing.

### Analysis of Sequencing Data

Unlike genome or exome sequencing, the nature of the V(D)J sequencing data from IgH locus requires some adjustments for reads mapping, as commonly used alignment software for sequencing data requires a reference sequence that is sufficiently similar to the sequences to be tested. As global alignment tools such as BWA [23] and Bowtie (version 1.0) [24] do not have optimal performance under default settings on reads spanning V(D)J junctions, we used BWA-SW [14], a local alignment tool, and found that it generally worked well to identify reads that match to V, V-D or V-D-J segments. For comparison, we utilized recently released IGBLAST mapping tool to explore V-(D)-J recombination events from the sequencing data. For somatic hypermutation analysis, we submitted the sequence data directly to the IMGT/GENE-DB [25] web server with several batches (due to the limitation of the web server to process large data sets), and analyzed the results generated by the web server.

### Accession Numbers

The raw sequencing data in the study are available for download from Sequence Read Archive (http://www.ncbi.nlm.nih.gov/sra) with project number **PRJNA213114**.
